# Clinical implications and utility of field cancerization

**DOI:** 10.1186/1475-2867-7-2

**Published:** 2007-03-15

**Authors:** Gabriel D Dakubo, John P Jakupciak, Mark A Birch-Machin, Ryan L Parr

**Affiliations:** 1 Genesis Genomics Inc., 310-1294 Balmoral Street, Thunder Bay, Ontario, P7B 5Z5, Canada; 2 National Institute of Standards and Technology, Biochemical Science Division, Gaithersburg, MD 20899, USA; 3 Dermatological Sciences, Institute of Cellular Medicine, Newcastle University, Newcastle upon Tyne, NE2 4HH, UK

## Abstract

Cancer begins with multiple cumulative epigenetic and genetic alterations that sequencially transform a cell, or a group of cells in a particular organ. The early genetic events might lead to clonal expansion of pre-neoplastic daughter cells in a particular tumor field. Subsequent genomic changes in some of these cells drive them towards the malignant phenotype. These transformed cells are diagnosed histopathologically as cancers owing to changes in cell morphology. Conceivably, a population of daughter cells with early genetic changes (without histopathology) remain in the organ, demonstrating the concept of field cancerization. With present technological advancement, including laser capture microdisection and high-throughput genomic technologies, carefully designed studies using appropriate control tissue will enable identification of important molecular signatures in these genetically transformed but histologically normal cells. Such tumor-specific biomarkers should have excellent clinical utility. This review examines the concept of field cancerization in several cancers and its possible utility in four areas of oncology; risk assessment, early cancer detection, monitoring of tumor progression and definition of tumor margins.

## Background

The sequential genetic changes that drive a cell towards malignancy occur over several years. In view of this, the American Association for Cancer Research Task Force on the treatment and prevention of intraepithelial neoplasia (IEN) has recognized the importance of targeting the treatment of early cancerous lesions to prevent or regress carcinogenesis [1]. Considerable research has identified molecular markers of IEN that serve as useful targets or endpoints of chemoprevention [1-3]. This laudable effort by the Task Force can be complemented by identification of biomarkers in normal tissues adjacent to tumors (peri-tumoral cancer fields). Validated biomarkers from cancer fields should be useful for primary chemoprevention studies as well.

The idea of field cancerization was conceived by Slaughter almost a decade prior to introducing the term in 1953. In an earlier publication he stated that; "*cancer does not arise as an isolated cellular phenomenon, but rather as an anaplastic tendency involving many cells at once*" [4]. The term "lateral cancerization" was subsequently used to indicate that the lateral spread of tumors was due to progressive transformation of cells adjacent to a tumor, rather than the spread and destruction of the adjacent epithelium by pre-existing cancer cells [5]. In a more extensive histopathologic review of 783 oral cancer patients, Slaughter and colleagues then used the term field cancerization to describe the existence of generalized carcinogen induced early genetic changes in the epithelium from which multiple independent lesions occur, leading to the development of multifocal tumors [6]. In some cases, multiple contiguous tumor foci coalesce that partly explain the lateral spread of squamous cell cancers. It was also observed that normal looking cells in close proximity to malignant cells were histologically abnormal and therefore were part of the transformed cells in a particular tumor field, and consequently were responsible for the occurrence of local tumor recurrences. These observations were made at about the era the DNA double helix was discovered by Watson and Crick, hence in the absence of modern molecular techniques. More recent studies using various genetic analyses have provided unequivocal evidence in support of the work of Slaughter and colleagues [7].

An important unanswered question of field cancerization is exactly how these cancer fields develop. Could a particular carcinogen induce multiple distinct genetic alterations in different cells resulting in polyclonal pre-neoplastic lesions in a particular tissue from which multiple tumors develop? Does a single genetic event occur in a cell that clonally expands and laterally spreads to replace the normal epithelium, and therefore create a large area of pre-neoplastic field from which multiple tumors develop? Alternatively, does an early genetic event occur simultaneously in a group of cells such that subsequent genetic lesions drive some cells towards malignancy? Is it also plausible that some field changes are created during organogenesis when some altered cells proliferate to generate a vast area of preconditioned epithelial surface, such that subsequent genetic lesions in some cells result in multiple cancers? Whereas all these modes of field cancerization are possible, molecular data accrued from studies of head and neck, esophagus, and bladder cancers support the clonal expansion model [8-10]. This model could explain how multiple tumors develop in organs such as skin, colon, esophagus, stomach, bladder, cervix, and vulva that have contiguous epithelium. Though possible, it is less likely that the spread of monoclonal cells is responsible for the development of multiple tumors in glandular tissues like the lung, breast, ovary, pancreas, and prostate that have discontinuous glandular epithelial organization, even when exposed to the same carcinogen. Multiple tumors in these organs are likely to originate from polyclonal tumor stem cells, although these clones may have identical genetic alterations. For example, loss of heterozygosity (LOH) in primary ductal carcinoma in situ (DCIS) were distinct from those in their corresponding cancers, suggesting that some DCIS and subsequent adenocarcinoma in the same breast developed from genetically divergent clones [11]. Furthermore, a study of microdissected normal glands and their co-existing malignant epithelia from breast cancer specimens demonstrated that only one had identical LOH to the cancer, with the majority harboring LOH at different genetic loci from the tumor [12].

Notwithstanding the mechanism of precancerous field development, the multistep model of tumorigenesis is probably better understood by field cancerization studies. Importantly, genetic information gained at various stages of cancer development continues to advance our knowledge and understanding of cancer biology, and how such molecular markers can be used clinically. Recent molecular techniques have enabled detection of alterations at all genomic levels. Epigenetic gene silencing, chromosomal anomalies, LOH, DNA sequence analysis for SNP and mutation detection, altered gene expression (transcripts and proteins), and mitochondrial genome changes have all been demonstrated in both precancerous lesions and normal appearing cells close to tumors [10,13-19]. One can even infer a metabolomic field cancerization profile in various tissues. It could be surmised that the early genetic changes will remain with the tumor cells as well as pre-neoplastic cells from the same organ. If well characterized, such early molecular alterations could have great value in risk assessment, early cancer detection, monitoring of disease progression, and chemoprevention.

A highly informative but almost forgotten genome is that contained in the mitochondrion. This organelle produces almost all the energy required for cellular physiology, and an inevitable byproduct of this reaction is the generation of reactive oxygen species (ROS), which can damage nucleic acids, proteins and lipids. The mitochondrial genome is less protected and has an inefficient repair mechanism compared to the nuclear genome, and yet is bathed by ROS. Not surprisingly therefore, the mitochondrial genome has a high mutation rate, and this may signal the genesis of cancer [20]. In addition, leakage of ROS into the nucleus can cause mutations in nuclear genes that could initiate the malignant process [21]. It is thus likely that the modest mtDNA molecule might sustain early genetic damage, and thus an even earlier biomarker indicative of field cancerization compared to nuclear genomic alterations that have been extensively studied.

Since the focus of this review is the identification and utility of genetic alterations in normal adjacent cells or precancerous cells that differ from normal donor control samples, the term field cancerization is used to broadly mean "the process whereby cells in a particular tissue or organ are transformed, such that genetically altered but histologically normal appearing cells predate the development of neoplasia or coexist with malignant cells, irrespective of clonality".

## Field cancerization is probably a general phenomenon of epithelial tumors

An important physiologic function of epithelia is their protective role that inevitably exposes them to environmental substances, including carcinogens that can create a vast area of genetically altered cancer fields. Epithelial cells frequently self renew and can undergo abnormal proliferation. Hyperplastic epithelia could form the basis of neoplastic transformation leading to the formation of the most common types of cancers of the human body. Molecular signatures of field cancerization have been documented for several epithelial tumors including those of the head and neck [22], esophagus [23], stomach [24], lungs [25], skin [26], cervix [27], vulva [28], bladder [29], colon [15], breast [17], ovary [30], pancreas [31], and recently the prostate [32]. It has even been conceptualized in brain and hematologic malignancies [33,34]. A brief synopsis of both nuclear and mitochondrial genetic markers used in field cancerization studies of several epithelial tumors is first provided.

### Head and neck cancer

Given that field cancerization was first described in oral cancers, it is not surprising that considerable molecular genetic studies have been conducted in head and neck tumors in an attempt to explain the mechanisms and importance of this phenomenon [7,35,36]. Whereas the debate on the clonal nature of these fields is ongoing, carefully designed experiments with strict definition of clonality (i.e., using cytogenetic markers, microsatellite instability, and mutation analysis) seem to support the notion that the majority of head and neck squamous cell carcinoma (HNSCC) originates from contiguous monoclonal pre-neoplastic fields [10]. Indeed a field size of over 7 cm has been mapped in these cancers [10], and about 62.5% of HNSCC second primary tumor recurrences are from similar clonal fields left behind after resection [13]. Recently, abnormal protein profiles were demonstrated in various samples of head and neck cancer in contrast to healthy patients. Profiling of mucosa from 73 healthy individuals, 113 HNSCC, 99 tumor-distant, and 18 tumor-adjacent discovered that 72% of tumor-adjacent and 27.3% of tumor-distance samples harbored aberrant protein profiles indicative of field cancerization. Interestingly, these altered protein profiles in tumor-distant samples were significantly associated with tumor relapse, indicating that proteomic analysis of peri-tumoral samples might have prognostic value [22]. It appears that carcinogen exposure creates a large molecularly altered pre-neoplastic field in the epithelium of the aerodigestive tract from which multiple tumors develop, and the remaining cancer fields left after resection give rise to local recurrences.

A role for geneomic alterations in stromal tissue in modulating the development of HNSCC was recently provided. Using laser-capture microdissection, tumor epithelial and stromal cells were obtained form 122 patients for whole genome LOH and allelic imbalance analysis using 366 microsatellite markers, and the results were compared to clinicopathologic parameters at presentation. Tumor size and nodal metastases were linked to three stroma-specific loci, whilst two epithelial loci were associated with nodal invasion [37]. Stromal genetic alterations therefore appear to control the growth and spread of HNSCC.

Field cancerization in HNSCC has also been addressed using mtDNA markers. Ha *et al*. [18] analyzed 137 premalignant lesions from 93 patients and demonstrated the presence of mtDNA C-tract alterations in 34 patients compared to their germline mtDNA. Notably, these mutations increased with increasing severity of dysplasia, suggesting acquired mitochondrial genome alterations might drive or indicate disease progression. Normal adjacent mucosas to dysplastic lesions were also analyzed. Identical mtDNA mutations were found in peri-lesional tissue of 3/8 lesions that had mtDNA alterations. Mutations persisted in 3/7 metachronous lesions, while 8/18 synchronous lesions had an identical pattern of mitochondrial mutations [18]. These findings lend support for the monoclonal nature of some head and neck cancer fields. In another study by this group, mitochondrial content alteration was observed in premalignant lesions and appear to increase with disease progression to malignancy [38]. These changes in mtDNA content can be detected in saliva [39], and the levels seem to decrease with treatment [40], indicating the possibility of a non-invasive means of early detection and monitoring of head and neck cancers using mtDNA markers. The levels of the 4977 bp common mtDNA deletion has been investigated by quantitative PCR in laser-capture microdissected tissues from paired oral cancers, their precancerous lesions and adjacent submucosal stroma in comparison to lymphocytes microdissected from lymph node biopsies [41]. The deletion was higher in lesions compared to lymphocytes. Interestingly, precancerous lesions had higher levels of the deletion than cancerous tissue, and in both cases, the adjacent submucosal stroma harbored more deletions than the lesions [41]. Thus the levels of the common deletion are increased in precancerous oral lesions, and appear to decrease with disease progression to invasive cancer. This might suggest transition to an advantageous energy producing pathway.

### Lung cancer

Smoking is an established carcinogen of lung cancer. In an attempt to map out smoking-related cancer fields, Franklin *et al*. [42] sampled tissues from the entire tracheobronchial tree of one individual who had 50-pack-years of smoking history without lung cancer, for *p53 *mutation analysis. They found that a transversion in codon 245 was present in 7/10 sites in both lungs, indicating a carcinogen targeted mutation in widespread lung epithelium of this individual [42]. Clone sizes in lung epithelium have been studied using twelve microsatellite markers on microdissected tumors and associated normal appearing bronchial epithelium. Clone sizes of up to 90,000 cells were identified [43]. Several sites of normal looking epithelium contained genetic abnormalities as well. Tumors from an individual tended to be homogeneous with respect to molecular markers, however, pre-neoplastic clonal patches were often heterogeneous [43]. More recently, LOH, especially at chromosome 12p12 was demonstrated in normal bronchial epithelium of long-term smokers, and deletion hotspots at two chromosomal regions (2q35-q36, 12p12p13) were observed in non-small cell lung cancer (NSCLC) and matched normal bronchial epithelial cells [25]. This suggests LOH could indicate susceptibility to or potential presence of cancer and may be a hallmark of progression of apparently phenotypically normal pre-neoplastic cells to cancer [44]. Thus, similar to the aerodigestive tract, it appears that carcinogens cause an early genetic change in widespread tracheobronchial epithelium from which multiple tumors develop. Given that about 72% of lung cancers diagnosed have already spread [45], non-invasive molecular profiling of sputum for biomarkers from tracheobronchial epithelial cancer fields in high risk population should aid early diagnosis.

### Esophageal cancer

Barrett's esophagus is a pre-neoplastic condition of esophageal cancer, and thus has served as a useful model for studies of clonality and tumor progression in field cancerization. Using *p53 *mutation as a clonal marker, Prevo *et al*. [8] mapped 213 endoscopic biopsies from 58 patients, and demonstrated that 50% were clonal and occupied cancer fields ranging from 1 cm to 9 cm [8]. LOH at 9p21 (*p16 *locus) and 17p13 (*p53 *locus) have also been used to map fields in the esophagus [46]. In 61 patients with 404 samples, LOH at one or both loci was observed in 73% of cases. Clone sizes were heterogeneous, with many clones showing incomplete expansion, however some were as large as 2 cm, and others occupied the entire Barrett's segment [46]. In another study, it was observed that clone expansion correlated with *p16 *status, being 1.5 cm in *p16+/+*, 6 cm in *p16+/-*, and 8 cm in *p16-/- *clones. Indeed, mutant *p16 *clones could expand to involve about 17 cm of the esophagus [23]. Epigenetic gene silencing of *APC*, *CDH1*, *ESR1 *and *p16 *has been studied in Barrett's esophagus. Hypermethylation in large contiguous fields were observed, further confirming molecular field cancerization in Barrett's metaplasia [47]. In a prospective study where 267 patients were followed, it was demonstrated that clone sizes (which is an index of clones multiplied by the length of Barretts's segment they occupied) with *p53 *LOH and ploidy, were a better predictor of progression towards adenocarcinoma [48]. Clones with *p16 *lesions did not carry much risk for tumor development in the absence of *p53 *LOH. Thus, clone expansion with added genetic instability seems to indicate disease progression in Barrett's esophagus. Recently, a clonal cellular diversity theory appears to explain progression of Barrett's esophagus towards adenocarcinoma. By applying the principles of ecology and evolution, (mutation rate, population size of evolving clones, rate of natural selection or clonal expansion), clonal diversity was a strong predictor of disease progression, even after controlling for known risk factors such as *p53 *LOH and ploidy abnormalities [49,50]. This finding is clinically relevant in terms of disease surveillance.

Recently, a new and sensitive array-based sequencing of mtDNA from 14 pre-neoplastic lesions of the gastrointestinal tract (7 Barrett esophagus, 4 colonic adenomas, and 3 inflammatory colitis-associated dyplasia) was performed. MtDNA mutations were observed in all 14 pre-neoplastic samples. Two colonic adenocarcinomas and the synchronous dysplastic lesions harbored identical genetic changes, suggesting a possible field cancerization [14]. This finding suggests that whole genome mtDNA profiling might help early detection of gastrointestinal tumors.

### Gastric cancer

Genetic evidence has been provided for the multicentricity of synchronous multiple gastric cancers. Analysis of mutation pattern in *APC*, *MCC *and *p53 *genes in multiple tumors from 13 patients (10 with double tumors, 2 with triple tumors and 1 with quadruple tumors), concluded that there was discordance in mutation patterns in these tumors [51]. Thus, independent genetic events in a preconditioned epithelium might have given rise to these multiple lesions. Epigenetic silencing via CpG island hypermethylation of *LIMS1*, a gene involved in cell dispersion has been demonstrated in 53% of gastric cancers [24]. Interestingly, *LIMS1 *methylation was observed in normal-appearing gastric tissue suggesting that this could be an early genetic event in the development of gastric neoplasia [24]. C-erb amplification was observed in a subset of tumors and the normal mucosa close to tumor margin [52], and aneuploidy was frequent in normal mucosa at about 3 cm distance from the tumor margin [53].

### Colorectal cancer

Colorectal cancers lend themselves to the study of field cancerization owing to the contiguous nature of their epithelium. Carcinoembryonic antigen (CEA) staining intensities in normal colonic mucosa adjacent to tumor was similar to that of the tumor, but this decreased at a distance of 1 cm, and staining at 5 cm from the tumor margin was identical to mucosa without tumor [54]. Thus, a gradient of CEA expression in peri-tumoral colon epithelium was observed. Recently, evidence for field cancerization in colorectal cancer has been provided by analysis of promoter methylation in the DNA repair gene, O^6^-methylguanine-DNA methyltransferase (*MGMT*) [15]. In this well designed study, methylation in tumors was observed to be associated with methylation in normal adjacent mucosa, and normal appearing colorectal mucosa located 10 cm away from tumors were methylated in 10/13 tumors. Normal mucosa located 1 cm from tumor margin was more likely to be hypermethylated than those 10 cm away [15]. Epigenetic silencing of *MGMT *thus creates a preconditioned genetic field from which colorectal tumors develop. Indeed, epigenetic events are proving to be useful biomarkers of the molecular process leading to colorectal cancer [55]. In another study, *K-ras *oncogene was found to be mutated and activated in 30% and 26% of adenomas respectively. Importantly, several downstream targets of *K-ras *were over-expressed in adenomas [56], indicating that these can be evaluated for early diagnosis and risk assessment.

### Vulval and cervical cancers

Vulval intraepithelial neoplasia (VIN) is often clonal and contiguous with vulval squamous cell carcinoma (VSCC). This suggests VIN may be a precursor lesion of VSCC. X-chromosome inactivation analysis of 9 cases of VIN, 10 cases of VSCC with contiguous VIN and 11 cases of VSCC with noncontiguous VIN indicates the majority of VIN and VSCC were monoclonal in origin. Two cases of VIN with noncontiguous VSCC, however, had molecular patterns consistent with separate clonal origins [28]. Allelotyping of three markers on chromosome 3p in microdissected samples from low and high grade cervical intraepithelial lesions found that microsatellite instabilities were common in low grade lesions associated with invasive cancers, suggesting pre-malignant and malignant lesions were of monoclonal origin [27]. Thus, biomarkers in early stage vulval and cervical lesions seem useful for early detection and monitoring of these cancers.

### Skin cancer

The organ most exposed to environmental carcinogens including ultraviolet radiation (UVR), is skin. Gene mutations caused by UVR play a critical role in the development of skin cancer. Different skin neoplasms are associated with signature gene mutations and alterations in gene expression. Precursor lesions such as actinic keratosis (AK) is associated with *p53 *mutations (and moderately increased *p16 *expression); squamous cell carcinoma (SCC) is associated with *p53 *mutations, increased *p16 *expression, activation of the mitogenic *ras *pathway, reduced expression of *FasR *(CD95-R) and increased expression of *FasL*; and basal cell carcinoma (BCC) is associated with mutations in *PTCH *(from the sonic hedgehog pathway) and *p53*. The contiguous nature and ease of accessibility of skin has made this organ suitable for studies of the mechanisms of how field cancerization develops. In fact, field cancerization in skin has been described as a process "whereby the whole neighborhood is affected". As described above, mutations in *p53 *are common in skin cancer and as such have been used as biomarkers of clonality in the skin. In one study, *p53 *mutations were present in non-melanoma skin cancer (NMSC) as well as the normal appearing peri-lesional skin of 8 patients [57]. Using whole-mount preparation, Jonason *et al*. [26] studied the clonal evolution and spread of *p53 *mutant keratinocytes arising from the dermal-epidermal junctions and hair follicles. Clones comprised of 60–3000 cells, and were larger and more frequent in sun-exposed than sun-shielded skin [26]. These genetically altered clones might await other genetic alterations to fully demonstrate the malignant phenotype. The carcinogenic effect of high dose therapy of psoriasis with UVA and psoralens is known. In a study of 69 tumors, *p53 *mutations were present in 54% of cases [58]. These mutant cells in multiple tumors from the same patient were heterogeneous, suggesting they arose from different somatic stem cell clones in cancer fields created by the UVA and psoralen treatment.

Corroborative evidence for field cancerization in the skin has been provided by mtDNA analysis. In NMSC, both tumors and the normal tissue adjacent to tumor (i.e., peri-lesional skin) contained homoplasmic UV-induced mtDNA mutations [59]. In another study, mtDNA deletions were present in both tumors and margin samples, with margin tissues harboring more deletions than tumor [60]. Thus, peri-lesional skin tissue might contain expanded mutant mtDNA keratinocytes, as has been demonstrated using nuclear DNA markers [26]. This suggests that the traditional use of histologically normal peri-lesional skin in NMSC studies may have several limitations. This is important when one considers that the majority of studies involving nuclear DNA damage and skin cancer/skin disease often use peri-lesional skin as a control tissue.

### Gallbladder cancer

In a well designed study, a panel of normal, pre-neoplastic and neoplastic gallbladder samples was analyzed for mitochondrial D310 alterations. Whereas D310 abnormalities were infrequent in normal samples, they increased in frequency in dysplastic lesions and normal appearing tissue adjacent to a tumor [61]. This finding calls for comprehensive analysis of mitochondrial genome alterations in pre-malignant gallbladder lesions.

### Bladder cancer

As the final recipient and reservoir of urine, the urothelium is inevitably exposed to carcinogens, which can create a large cancer field in this tissue resulting in multifocal tumors. Whole organ mapping of bladder cancer fields has been studied using a combination of LOH, *p53 *mutation and fluorescence in situ hybridization (FISH) analysis [62]. Cells were microdissected from various parts of a cystectomised bladder for analysis. Several abnormalities not observed in normal bladder were present in bladder with cancer. Molecular and histopathologic data comparison suggested monoclonality of multifocal lesions in bladder cancer [62]. In another investigation, 32 tumors from 6 bladders were analyzed using chromosomal markers [9]. Interestingly, multiple tumors from the same bladder harbored identical chromosomal alterations in addition to private chromosomal changes, as is expected of the multistep model of tumorigenesis. Indeed it is likely that in the bladder, lesions occur in genetically transformed but histologically normal urothelium [63].

### Breast cancer

Glandular epithelial cells of the breast undergo cyclical proliferation, which favors neoplastic transformation. DCIS is a precancerous lesion of invasive breast cancer. Several groups have demonstrated genomic instability in normal breast lobules adjacent to cancer focus, and in DCIS. LOH in normal breast tissue adjacent to breast cancer was reported in 8/30 cases, all of which possessed the same missing allele as the corresponding carcinoma [16]. Analysis of LOH was performed on fine needle aspiration (FNA) biopsy samples obtained from 30 asymptomatic (11 with normal cytology and 19 with proliferative cytology) women with known risk of breast cancer. LOH was observed in 2 and 14 patients with normal cytology and abnormal cytology respectively [64]. These findings suggest that random FNA biopsy sampling of breast tissue for molecular screening could potentially be useful in individualized medicine. In another study, normal appearing tissue samples were obtained from breast quadrants of 21 patients with known breast cancer for studies of LOH and allelic imbalances [65]. Genomic instabilities were higher in outer breast quadrants than inner quadrants. Thus, the increased frequency of breast cancer in outer quadrants is related to the presence of elevated genomic instabilities [65]. LOH was also studied on samples from 30 women with grade 1 and grade 3 DCIS, and 6 patients who subsequently developed invasive cancer [11]. At four chromosomal loci (6q, 11p, 17p and 17q), there were higher losses in grade 3 compared to grade 1 DCIS, however, the fractional allelic loss at 19 loci was significantly higher in grade 1 than in grade 3 DCIS. As well, LOH in DCIS and corresponding invasive breast cancer were heterogeneous [11]. On the contrary, a previous study had indicated the possible progressive nature of DCIS to invasive ductal carcinoma (IDC) using similar LOH analysis. In this study, samples from 7 women with DCIS who subsequently developed breast cancer in the same breast were examined. At 50 loci, LOH in DCIS and IDC were concordant, and LOH appeared to accumulate with disease progression from DCIS to IDC [66]. Alterations in telomere DNA content and allelic imbalances were demonstrated in histologically normal breast tissue located at 1 cm distance from visible tumor margin. These changes decrease as a function of distance from the tumor margin [17].

Epigenetic gene silencing in breast cancer has been studied. Methylation of the *cyclin D2 *promoter appeared to be specific to breast cancer, however, promoter methylation of *APC*, *RARbeta2 *and *RASSF1A *in normal breast from breast cancer patients was associated with increased breasts cancer risk [67]. In a recent comprehensive study of methylation of *RASSF1A *promoter in breast tissue samples, it was uncovered that primary tumors had significantly higher promoter methylation than control reduction mammoplasty tissue, with adjacent normal samples having intermediate levels. Interestingly, global profiling of DNA methylatation revealed more methylated genes in normal adjacent samples than in normal donor control samples [68].

### Prostate cancer

Compared to breast, relatively little work has been conducted on field cancerization in prostate cancer. Prostate cancer is often multifocal, and it is likely that multiple tumors arise from an organ genetically altered by a particular carcinogen. Genomic instability, gene expression studies and analysis of mitochondrial genome alterations have recently been reported to show field cancerization in prostate cancer. Methylation in *GSTP1 *and *RARbeta2 *was present in prostate cancer, adjacent stroma and adjacent normal glands close to tumor and were absent in normal epithelia from benign prostatic hyperplasia [32]. Telomere content alterations were observed in normal appearing tissue close to tumors, and was shown to be a good predictor of prostate cancer recurrence [69]. Injection of prostate cancer cell lines into athymic nude mice caused cytogenetic abnormalities in stromal cells [70], suggesting that at least in prostate cancer, tumor cells might have the potential of transforming adjacent normal glands. Gene expression signatures in normal tissue adjacent to a tumor focus closely resembled those of tumors and were different from normal donor prostate samples [19,71]. Interestingly, early prostate cancer antigen (EPCA), which is expressed in normal tissues close to a tumor, was shown to have elevated expression in normal prostate glands of individuals who subsequently developed cancer years later [72,73]. The possible clonal nature of multifocal prostate cancer is suggested by a recent study. Laser-capture microdissection was used to procure pure glandular epithelial cells from multifocal tumors for gene expression studies. In any particular individual, *ERG, ETV1*, and *ETV4 *were either over-expressed or not expressed in all samples, suggesting alteration in these genes could be early events in prostate cancer evolution [74]. Prostate cancer is an age-associated disease. Interestingly, a prostate cancer DNA phenotype, likely induced by an age-associated oxidative DNA damage, was found in some older men and in normal prostate glands adjacent to tumors [75,76]. This cancer DNA conformation is likely an early event in prostate cancer development since it occurs long before tumors develop [77]. Indeed a metastatic prostate cancer DNA phenotype was also demonstrable in metastasizing tumors and the normal glands surrounding these tumors, and this phenotype was different from that of primary cancer phenotype [78]. Some of the molecular changes in prostate cancer appear to have excellent potential utility as early diagnostic biomarkers [79].

Although not directly questioned, the concept of field cancerization is indicated in a number of mtDNA mutation studies of prostate cancers. Mutations in the mitochondrial genome were present in a co-existing precursor lesion, PIN and matched tumor [80]. In a comprehensive study of laser capture microdissected tissues from prostatectomy specimens, we demonstrated some aspects of field cancerization using mitochondrial genome markers [81]. Pure populations of cells from a malignant focus and the normal appearing cells at two distinct locations, immediately proximal to the tumor and further away from the tumor were obtained. MtDNA mutation load in tumors and matched normal appearing glands were identical and these were significantly different from those obtained from age-matched control individuals without cancer [81]. In a follow-up study, a single large scale mtDNA deletion associated with prostate cancer was observed to increase in frequency in normal appearing glands adjacent to a tumor.

### Ovarian cancer

The most common ovarian cancer, serous ovarian carcinoma, can develop following serous borderline ovarian tumors (BOTs), suggesting the ovarian epithelium could be genetically preconditioned by a carcinogen from which tumors develop. Moreover, it has been the conception that multifocal and recurrent ovarian tumors are monoclonal. A study of clonality using *p53 *and *K-ras *mutation analysis in 8 patients with BOTs who later developed serous carcinomas concluded that the tumors were unrelated [82]. Likely, distinct genetic alterations in a preconditioned epithelium may lead to BOTs and serous carcinomas independently. Analysis of 13 primary and corresponding recurrent ovarian tumors using four markers of genomic instability indicated 10/13 were different, with the rest being identical [83]. Promoter methylation status of *hMLH1*, *CDKN2A*, and *MGMT *in synchronous independent ovarian and endometrial cancers was studied for the possible origin of these tumors from a cancerization field. High frequency of promoter methylation of *CDKC2A *and *MGMT *were found in both endometrial and ovarian carcinomas, suggesting the epigenetic silencing of these genes could be an early event in the development of these synchronous cancers [30].

### Pancreatic cancer

Multiple intraductal papillary tumors of the pancreas are common, and a precancerous lesion of pancreatic adenocarcinoma is ductal hyperplasia. Therefore could pancreatic cancers arise from genetically transformed but normal ductal epithelium? Mutations in *K-ras *are early important events in pancreatic ductal carcinoma and non-neoplastic pancreatic ductal lesions. Therefore, these markers have been used for several studies of pancreatic cancer evolution. Microdissected tumors and associated ductal hyperplastic tissue from 37 patients for *K-ras *mutation and X-chromosome inactivation analyses demonstrated distinct genomic abnormalities in hyperplasia as well as pancreatic cancer. This study concluded that polyclonal multicentric pancreatic cancers originate form epithelium with early genetic changes [84]. In a study of microdissected samples from 20 intraductal papillary-mucinous tumors (IPMT) and 7 ductal adenocarcinoma, *K-ras *mutations were noted in 66.7% of peri-tumoral tissue and 62.5% of separate IPMT lesions, and at least one identical mutation was observed in the tumor and peritumoral tissue in all IPMT patients with those lesions [31]. *K-ras *mutation as a marker of disease progression has been demonstrated. Analysis of 46 different histologic grades of IPMT from 16 patients and 9 with ductal adenocarcinoma reveal an increased frequency of *K-ras *mutation from 16.7% in normal epithelium and papillary hyperplasia, to 57.1% in high grade dysplasia, carcinoma in situ and invasive cancer [85].

## Conceptualization of field cancerization in other tumors

### Hematologic oncology

It has been proposed that generalized insults to bone marrow can lead to simultaneous generation of many abnormal bone marrow clones. Therefore admixture of cells representing severe aplastic anemia, acute promyelocytic leukemia, chronic myeloid leukemia, and myelodysplastic syndrome, can be present in the same bone marrow, and that proliferative advantage of one clone results in its dominant appearance and thus specific diagnosis. The term "field leukemogenic effect" was used to describe this phenomenon [33]. It will be interesting to know whether the ecological and evolutionary concepts used to predict progression in Barrett's esophagus to adenocarcinoma [49] applies here as well.

Heteroplasmy is an early indicator of disease, and in oncology, this could probably be an indicator of field cancerization. The dynamics of mitochondrial genome changes was shown in association with the progression of myelodysplastic syndrome (MDS) to acute myeloid leukemia (AML) [86]. MDS are clonal myeloid disorders that can transform to AML. A heteroplasmic mtDNA mutation in MDS was observed to increase with disease evolution until the final stage of AML when the mutant copy became homoplasmic. This sequencing data was confirmed using restriction digest analysis by showing that the mtDNA mutation load positively correlated with progression from MDS to AML [86]. These findings are indeed very interesting, and importantly emphasize the need for sensitive sequencing methods for heteroplasmy detection.

### Neuro-oncology

Gliomatosis cerebri (GC) is a rare neoplasm of the brain with extensive distribution, usually involving both lobes and even occasionally infratentorial regions. Could this lesion therefore arise from a vast field of preconditioned neural tissue? In one study of GC, 24 tissue samples were randomly obtained from several brain areas for study. Genome-wide scan for chromosomal aberrations, *p53 *mutation analysis and LOH were performed on all samples. Mutations in *p53 *were present in 20/24 cases, with chromosomal loses and allelic imbalances in several other tumor samples [34]. In a separate series, mtDNA was used as a clonal marker for GC, and consistent band losses were observed in all tumor samples from two individuals, one of who also had *p53 *mutations [87].

## The need for appropriate control tissue in cancer studies

An important message from the above review of the literature is that using histologically normal appearing samples as the sole control tissue in cancer research is probably inappropriate [88]. At its minimum, the use of donor tissues (ideally obtained under similar conditions as tumor) will serve as better controls for tumor-specific biomarker discovery. Donor control, in addition to normal adjacent to tumors, precancerous lesions, and tumor samples will provide the best sample set for resolution of genetic alterations that are relevant to the disease process. If normal adjacent to tumor tissue must be used as the only normal control, it should first be examined for the absence of genetic abnormalities [88]. In skin cancer studies for example, additional analysis of UV-induced damage in sun protected skin from the same individual would provide a complementary isogenic control. With regards to mtDNA analysis, it is regrettable that a number of investigators are still using normal appearing cells in the presence of tumors as normal maternal germline control tissue from which somatic mtDNA mutations could be unraveled [89-97]. Obviously this will lead to erroneous mutations being associated with the disease processes, since the adjacent normal tissue in a cancerization field may sustain somatic mtDNA mutations as well. Comparison to the mtDNA profile of the individual's blood, which we have determined to be the authentic germline mitochondrial genome, is necessary.

## Clinical implications

### Risk assessment, early detection, chemoprevention, and disease progression

Biomarkers present in tumors and not in normal appearing cells close to a tumor are thought to be useful for early detection of cancer. Usually such tumor-specific biomarkers are validated and used for screening to diagnose organ confined tumors that have better treatment outlook. In some instances, early detection strategies such as molecular profiling of circulating tumor cells rather indicate the presence of metastasis [98]. Future biomarker discovery and validation efforts should focus on identification of biosensors that signal the genesis of disease, rather than biomarkers of the disease. Such biosensors will be useful in risk assessment, early detection, disease monitoring, and chemoprevention. For example, LOH in normal breast epithelial cells obtained by random FNA biopsy from women with known risk of breast cancer was used to predict breast cancer risk [64]. In this study, the Gail risk model predicted a mean lifetime breast cancer risk of 16.7% for women with no LOH compared to 22.9% for women with LOH [64]. These markers thus correlate with individual risk of developing breast cancer, and thus seem useful for early detection and risk assessment of breast cancer. Genetic changes present in normal appearing cells can be used for identification and recruitment of individuals at risk of developing cancer for primary chemoprevention (i.e., to prevent de novo development of cancers). Indeed, epigenetic gene silencing through promoter hypermethylation and transcriptional repression of several tumor-associated genes is an early event in several cancers including breast, prostate, colorectal, gastric, and ovarian cancers. Importantly, reversal of epigenetic events with agents such as hydralazine, 5-Aza-2'-deoxycytidine, zebularine, and magnesium valporate is possible. Knowledge of methylation patterns and their role in malignant transformation will enable controlled use of mehtylation reversal agents in primary chemoprevention. Similarly, relevant markers present in precancerous lesions will be useful endpoint measures or targets of secondary chemoprevention (i.e., to prevent the progression of pre-malignant lesions to invasive cancers). Importantly, biofluids, representative of cells from a particular organ may serve as useful noninvasive or minimally invasive samples for disease surveillance. For instance, genetic changes preceding breast cancer development might be detectable in nipple aspirate fluids.

Multistep field cancerization indicates two levels of cancer progression: *molecular progression *whereby histologically normal looking cells undergo sequential cumulative acquisition of genomic damage, and *phenotypic progression *whereby a neoplastic cell accumulates genetic alterations and undergoes further phenotypic changes (e.g., from intraepithelial neoplasia to various stages of invasive cancer). Functionally relevant pathways altered at the molecular progression phase should uncover useful biosensors for early detection and monitoring of cancer. It is also well known that not all precancerous lesions progress to invasive cancers [1]. Thus, molecular profiling of early lesions using appropriate control tissue will enable important pathways or biomarkers that predict disease progression to be deciphered. In a recent elegant study, laser-capture microdissection was used to procure pure population of cells at various stages of prostate cancer development for gene expression profiling. Using a novel analytical approach referred to as "molecular concept model", several genes and pathways were identified that represented molecular progression of prostate cancer from benign through prostatic intraepithelial neoplasia to prostate cancer. Increased expression of genes involved in cell cycle regulation, and on 8q was associated with disease progression [74]. The estimated progression of an atypical hyperplasia to an adenocarcinoma may occur over a period of 5–20 years. This prolonged time course provides opportunities for early detection within an activated pre-cancerous epithelial field [1,2].

An important clinical utility of field cancerization is in complementary evaluation of pathologic biopsy specimen. Currently, biopsies for cancer diagnosis are reviewed by histology, the gold standard, and the absence of abnormal cells often precludes the diagnosis of cancer. However, histologically normal biopsy specimen that possess molecular signatures of cancer fields suggest either the tumor was missed by the biopsy procedure, or that some cells in the tissue are progressing towards malignancy. Such high risk patients will require close surveillance for early detection of disease.

### Tumor margins and recurrences

Tumor recurrences are common in surgical oncology. Depending on the type of tumors, recurrent rates could be as high as 50% [99]. There are two types of local tumor recurrences; those that occur at the primary site of surgical recurrence (local or scar recurrence), and those that occur at a distance from the surgical scar in the residual organ left after resection of the primary tumor (*in situ *recurrence, second primary tumor (SPT) or second field tumors (SFT)). *In situ *recurrences that are genetically similar to the primary tumor are referred to as SFT, and those that are genetically dissimilar to the primary are identified as true SPT. Field cancerization may have an etiologic role in a substantial number of recurrences. For example, a surgical resection margin that includes a genetically altered field can explain the occurrence of scar recurrence. This explanation suggests that molecular profiling of surgical margins will help reduce scar recurrences. Since multiple independent patches of cancer fields may be present in the same organ exposed to the same insult, clean molecular margins may not necessarily prevent recurrences in the residual organ. Nonetheless, complementary molecular assessment of tumor margins should at least help reduce tumor recurrences. For instance, in pancreatic cancer, analysis of *K-ras *codon 12 mutation in histologically normal surgical margin tissues from 70 patients could have prognostic significance. In this study, 53% of patients with positive molecular margins had unfavorable overall survival outcome [100].

## Conclusion

Field cancerization is a well known and well documented process of malignant transformation. Several studies confirm the importance of this phenomenon in tumor development. With technological advancement, the future should benefit from well designed studies aimed at identifying genetic markers and pathways useful in disease management. An obvious shortcoming in almost all the studies of field cancerization is the lack of extensive genome-wide scans that will enable early and important genetic changes in tumor evolution to be uncovered. Many studies have relied heavily on known markers associated with a particular tumor. Such selected tumor markers might be later acquisitions in the disease process, and will be missed in peri-tumoral samples or precancerous lesions. Comprehensive high-throughput analyses for the discovery of early and relevant genetic changes that extend across global networks and represent modular alterations of multiple targets (or surrogates) of terminal histologically differentiated stages of cancer subtypes will be essential for early detection, risk assessment and primary chemoprevention.

## Competing interests

The author(s) declare that they have no competing interests.

## Disclaimer

Certain commercial equipment, instruments, materials or companies are identified in this paper to specify adequately the experimental procedure. Such identification does not imply recommendation nor endorsement by the National Institute of Standards and Technology, nor does it imply that the materials or equipment identified are the best available for the purpose.
